# Diversity of *Plectosphaerella* within aquatic plants from southwest China, with *P. endophytica* and *P. sichuanensis* spp. nov.

**DOI:** 10.3897/mycokeys.80.64624

**Published:** 2021-05-11

**Authors:** Xiao Qian Yang, Shi Yun Ma, Ze Xiang Peng, Zhong Qiao Wang, Min Qiao, Zefen Yu

**Affiliations:** 1 Laboratory for Conservation and Utilization of Bio-resources, Key Laboratory for Microbial Resources of the Ministry of Education, Yunnan University, Kunming, Yunnan, 650091, China Yunnan University Kunming China; 2 Tianma development office of Yiliang county, Yunnan Province, China Tianma development office of Yiliang county Zhaotong China

**Keywords:** Endophytic fungi, multi-locus phylogeny, new species, Plectosphaerellaceae, Sordariomycetes

## Abstract

Members of *Plectosphaerella* inhabit different substrates, including plants, soil and insects, and most species are pathogens causing large losses in agriculture. During a survey of endophytic fungi in aquatic plants in southwest China, 112 strains of *Plectosphaerella* were isolated, representing two new species, *P. endophytica***sp. nov.** and *P. sichuanensis***sp. nov.**, as well as two known species, *P. cucumerina* and *P. pauciseptata*. The novel taxa are described and illustrated here using combined morphological and multi-locus phylogenetic (LSU-ITS-TEF-1α-TUB2) analyses. Our result revealed *Plectosphaerella* species inhabiting within aquatic plants in southwest China, and the separation frequency of each species was presented.

## Introduction

The genus *Plectosphaerella* Kleb. was established to accommodate *P. cucumeris* Kleb. from young cucumber plants (Klebahn 1929). Previously, it was always placed in Hypocreaceae (Sordariomycetes, Hypocreales) and Sordariaceae (Sordariomycetes, Sordariales) (Domsch and Gams 1972; Uecker 1993), until Zare et al. (2007) established Plectosphaerellaceae W. Gams, Summerb. & Zare (Glomerellales) to accommodate it (Réblová et al. 2011). The genus *Plectosporium* Palm, Gams & Nirenberg was described as the asexual morph of *Plectosphaerella* (Palm et al. 1995). Given its priority, *Plectosphaerella* was recommended as the accepted generic name (Réblová et al. 2016), and all species of *Plectosporium* were transferred into *Plectosphaerella* (Carlucci et al. 2012). At present, there are 23 species records for *Plectosphaerella* as listed in Index Fungorum (2021), two species, *P. himantia* (Pers.) Kirschst. and *P. melaena* (Fr.) Kirschst. are not recognized for *Plectosphaerella*.

Species of *Plectosphaerella* are distributed in a variety of habitats and have wide geographic distribution (Carlucci et al. 2012; Zhang et al. 2019). Some species are pathogens on the fruit, root or collar in Cucurbitaceae plant, causing large losses of melon, pumpkin and zucchini crops (Alfaro-García et al. 1996; Toyozo et al. 2005; Raimondo and Carlucci 2018). Several species harm other plants such as tomato, pepper, bamboo, and asparagus. And the symptoms of hosts are decomposition, breakdown and death (Antignani et al. 2008; Carlucci et al. 2012; Arzanlou et al. 2013). Besides diseased plants, *P. sinensis* Lei Su & Y.C. Niu was reported as endophytic fungus without causing obvious symptoms (Su et al. 2017). In addition, most species causing plant diseases were also isolated from soil, except for *P. oratosquillae* (P.M. Duc et al.) A.J.L. Phillips et al. originating from arthropod *Oratosquilla oratoria* (Duc et al. 2009).

*Plectosphaerella cucumerina* is the most widely distributed species, thriving on more than nine plant genera: *Arabidopsis*, *Cucumis*, *Galium*, *Hydrilla*, *Nicotiana*, *Pyrus*, *Solanum*, *Viola* and *Austropotamobius* etc., and also occurring in soil and paper (Alderman and Polglase 1985; Palm et al. 1995; Smith-Kopperl et al. 1999; Domsch et al. 2007; Giraldo and Crous 2019). Meanwhile, this species has a wide geographic distribution, and it has been reported from England, Italy, Canada, the Netherlands, Egypt etc. (Raimondo and Carlucci 2018, Giraldo et al. 2019). The second species with wide geographic distribution, *P. plurivora* A.J.L. Phillips et al., was found in Australia, the Netherlands, Germany, USA etc. Its hosts include *Lolium*, *Solanum*, *Nicotiana*, *Asparagus* and it also occurs in soil (Carlucci et al. 2012; Giraldo and Crous 2019). However, other species did not show obvious habitats diversity and wide geographic distribution.

During our investigation of endophytic fungal diversity of aquatic plants in southwest China, among 1697 acquired strains, 112 strains belonging to *Plectosphaerella* were isolated. Based on morphological characteristics and phylogenetic analysis, two known species, *P. cucumerina* and *P. pauciseptata*, were described, and two new species, *P. endophytica* and *P. sichuanensis*, were proposed and illustrated. The geographic distribution and habitat diversity of *Plectosphaerella* in this study were also discussed.

## Materials and methods

### Isolates and Morphology

Samples were collected from Yunnan, Guizhou, Sichuan provinces, Chongqing and Tibet from 2014 to 2017. The dominant hosts are *Myriophyllum*, *Potamogeton*, *Hydrilla* and *Hippuris*. Samples were placed in plastic bags, labeled and transported to the laboratory. Each leaf and stem was cut into segments 30–40 mm in length and washed thoroughly with tap water, then a surface-disinfection was carried out according to Su et al. (2016). The segments were cut into smaller sections of about 5×5 mm under the aseptic operating table. Firstly, washed 30 s in sterile water and soaked 2 min in 0.5% hypochlorite solution, then 30 s in sterile water and 2 min in 75% ethanol, finally 30 s in sterile water. Each ten sections were randomly isolated on rose bengal agar (RBA, Guangdong Huankai Microbial Sci and Tech), the antibiotics chloramphenicol (0.1 g l^–1^) was added to restrain bacterial growth. When a fungus grew up from the segments, some hyphae were picked up and transferred to potato dextrose agar (PDA, 200 g potato, 20 g dextrose, 18 g agar, 1000 ml distilled water) plates for incubation at 28 °C. After 10 days, colonies were transferred to different plates, including corn meal agar (CMA, 20 g cornmeal, 18 g agar, 1000 ml distilled water) and oatmeal agar (OA, 30 g filtered oat flakes, 20 g agar, 1000 ml distilled water). Colony characteristics, growth speed and other macrostructure from PDA plates were observed after 10 days. Microscopic characteristics such as mycelium, conidiophores and conidia were examined and measured after 3 days on CMA using BX51 microscope (Olympus); the sterile water was used as a mounting medium.

Pure cultures were deposited in the Herbarium of the Laboratory for Conservation and Utilization of Bio resources, Yunnan University, Kunming, Yunnan, P.R. China (YMF, formerly Key Laboratory of Industrial Microbiology and Fermentation Technology of Yunnan) and at the China Center for Type Culture Collection (**CCTCC**).

### DNA extraction, PCR amplification and sequencing

Actively growing mycelium was scraped off from the surface of the culture and transferred to 2 ml Eppendorf micro-centrifuge tubes. Total genomic DNA was extracted follow the protocol of Guo et al. (2000). The internal transcribed spacer (ITS) and the 28S large subunit nuclear ribosomal RNA (LSU rRNA) were amplified using the primer pairs ITS1/ITS4 (White et al. 1990) and LROR/LR5 (Vilgalys and Hester 1990), respectively. Translation elongation factor 1-alpha (TEF-1α) and partial β-tubulin (TUB2) were amplified using the primer pairs EF-1251R/EF-688F (Alves et al. 2008) and Bt2a/Bt2b (Glass and Donaldson 1995), respectively. The PCR amplifications were conducted in 25 µl final volumes which consisted of 1.0 µl DNA template, 1.0 µl of each forward and reverse primers, 12.5 µl 2 × Master Mix and 9.5 µl ddH_2_O. The PCR reaction cycles were as follows: initial denaturation at 94 °C for 3 min; followed by 35 cycles of denaturation at 94 °C for 40 sec; the annealing extension dependent on the amplified loci (48 °C for LSU, 54 °C for ITS, 55 °C for TEF-1α and 58 °C for TUB2) for 1 min and extension at 72 °C for 2 min; a final extension at 72 °C for 10 min. PCR products were sequenced by TSINGKE Biological Technology in Kunming, China. The sequences are deposited in GenBank database and the accession numbers are listed in Table 1.

**Table 1.** *Plectosphaerella* species used in phylogenetic analyses.

Species	Strain no.	GenBank accession no.			
		LSU	ITS	TEF-1α	TUB2
*Monilochaetes infuscans*	CBS 379.77	GU180645	LR026764	–	–
*Plectosphaerella alismatis*	CBS 113362^T^	KY662261	KY399815	KY421328	KY421304
*P. citrullae*	CBS 131740	KY662254	KY399821	KY421319	KY421307
	CBS 131741^T^	KY662255	KY399822	KY421320	KY421308
*P. cucumerina*	3F17	KY399831	KY401602	KY421331	KY421300
	3F24	KY399832	KY401603	KY421332	KY421301
	3F42	KY399829	KY401604	KY421330	KY421299
	3F43	KY399830	KY401605	KY421334	KY421302
	CBS 131739	KY662258	KY399816	–	–
	CBS 137.37	KY662256	KY399827	–	–
	**YMF 1.04692**	**MW326671**	**MW024075**	**MW441776**	**MW441782**
*P. delsorboi*	CBS 116708^T^	EF543843	KY499237	KY499238	KY499240
***P. endophytica***	**YMF 1.04701**	**MW024052**	**MW024054**	**MW029607**	**MW029608**
*P. guizhouensis*	CGMCC 3.19658^T^	MK880431	MK880441	–	–
	CGMCC 3.19659	MK880432	MK880442	–	–
	CGMCC 3.19660	MK880433	MK880443	–	–
*P. hanneae*	CBS 144925	LR590378	LR590201	–	–
*P. humicola*	CBS 423.66^T^	LR025949	LR026811	–	–
*P. kunmingensis*	KUMCC 18-0181	MK993015	MK993014	–	–
*P. melonis*	CBS 489.96^T^	KY662260	KY399813	KY421329	KY421295
	CBS 409.95	KY662259	KY399814	–	KY421296
*P. nauculaspora*	CGMCC 3.19656^T^	MK880424	MK880439	–	–
	CGMCC 3.19657	MK880425	MK880440	–	–
*P. oligotrophica*	CGMCC 3.15078^T^	JX508806	KY399817	KY421315	KY421309
	CGMCC 3.15079	JX508807	KY399818	KY421316	KY421310
*P. oratosquillae*	NJM 0662^T^	–	AB425974	–	–
	NJM 0665	–	AB425975	–	–
*P. pauciseptata*	CBS 131744	KY662251	KY399824	KY421322	–
	CBS 131745^T^	KY662250	KY399823	KY421321	–
	**YMF 1.05088**	**MW326672**	**MW024087**	**MW441777**	–
	**YMF 1.04679**	**MW326673**	**MW024105**	**MW441778**	**MW441783**
	**YMF 1.04725**	**MW466575**	**MW024104**	**MW441779**	**MW441784**
*P. populi*	CBS139623^T^	KY662257	KY399828	KY421336	KY421311
	CBS 139624	KR476784	KR476751	–	–
*P. plurivora*	CBS131742^T^	KY662248	KY399826	KY421324	KY421298
*P. ramiseptata*	CBS 131743	KY662252	KY399819	KY421318	KY421306
	CBS 131861^T^	KY662253	KY399820	KY421317	KY421305
***P. sichuanensis***	**YMF 1.05081**	**MW027535**	**MW027539**	**MW309880**	**MW441780**
	**YMF 1.05082**	**MW027536**	**MW027534**	**MW441775**	**MW441781**
*P. sinensis*	ACCC 39144	KX527892	KX527889	KY421326	KY421313
	ACCC39145^T^	KX527891	KX527888	KY421325	KY421312
	ACCC 39146	KX527893	KX527890	KY421327	KY421314
*P. verschoorii*	CBS 144924^T^	LR590476	LR590240	–	–
	JW 13006	LR590415	LR590241	–	–
	JW 62006	LR590426	LR590252	–	–

Strains and sequences generated in this study are emphasized in bold face. ^T^ex-type cultures.

### Phylogenetic analyses

The ITS sequences generated in this study were used as a query to search similar DNA sequences using BLASTn. All published DNA sequences were obtained from the GenBank from relevant studies (Su et al. 2017; Phookamsak et al. 2019; Zhang et al. 2019), *Monilochaetes infuscans* Harter (Australiascaceae) was selected as an outgroup. The generated sequences were manually aligned with CLUSTAL_X v. 1.83 (Thompson et al. 1997) with default parameters. Aligned sequences of multiple loci were concatenated and manually adjusted through BioEdit version v. 7.0.4.1 (Hall 1999), and ambiguously aligned regions were excluded. The combined sequence was converted to a NEXUS file using MEGA6 (Tamura 2013). The alignment was deposited at TreeBase http://purl.org/phylo/treebase/phylows/study/TB2:S27363.

Maximum-likelihood (ML) analysis was conducted by using RAxML (Stamatakis 2006) with the PHY files generated with CLUSTAL_X v. 1.83 (Thompson et al. 1997), using the GTR+GAMMA model. ML bootstrap proportions (MLBPs) were computed with 1000 replicates. Bayesian inference (BI) analysis was executed with MrBayes v. 3.2.2 (Ronquist and Huelsenbeck 2003). The Akaike information criterion (AIC) implemented in jModelTest version 2.0 was used to select the best fit models after likelihood score calculations (Posada 2008). HKY+I+G was estimated as the best-fit model under the output strategy of AIC, Lsetnst=6, rates=gamma. A Markov Chain Monte Carlo (MCMC) algorithm was used to generate phylogenetic trees with Bayesian probabilities. Two runs were executed simultaneously for 6,000,000 generations and sampled every 500^th^ generations, four chains containing one cold and three heated were run until the average standard deviation of the split frequencies dropped below 0.01, the stationarity of the analyses was confirmed in line with standards described by Sun and Guo (2010). The initial 25% of the generations of MCMC sampling were discarded as burn-in. The refinement of the phylogenetic tree was used for estimating BI posterior probability (BIPP) values. The tree was viewed in FigTree version 1.4 (Rambaut 2012).

## Results

### Phylogenetic analyses

**Figure 1.**
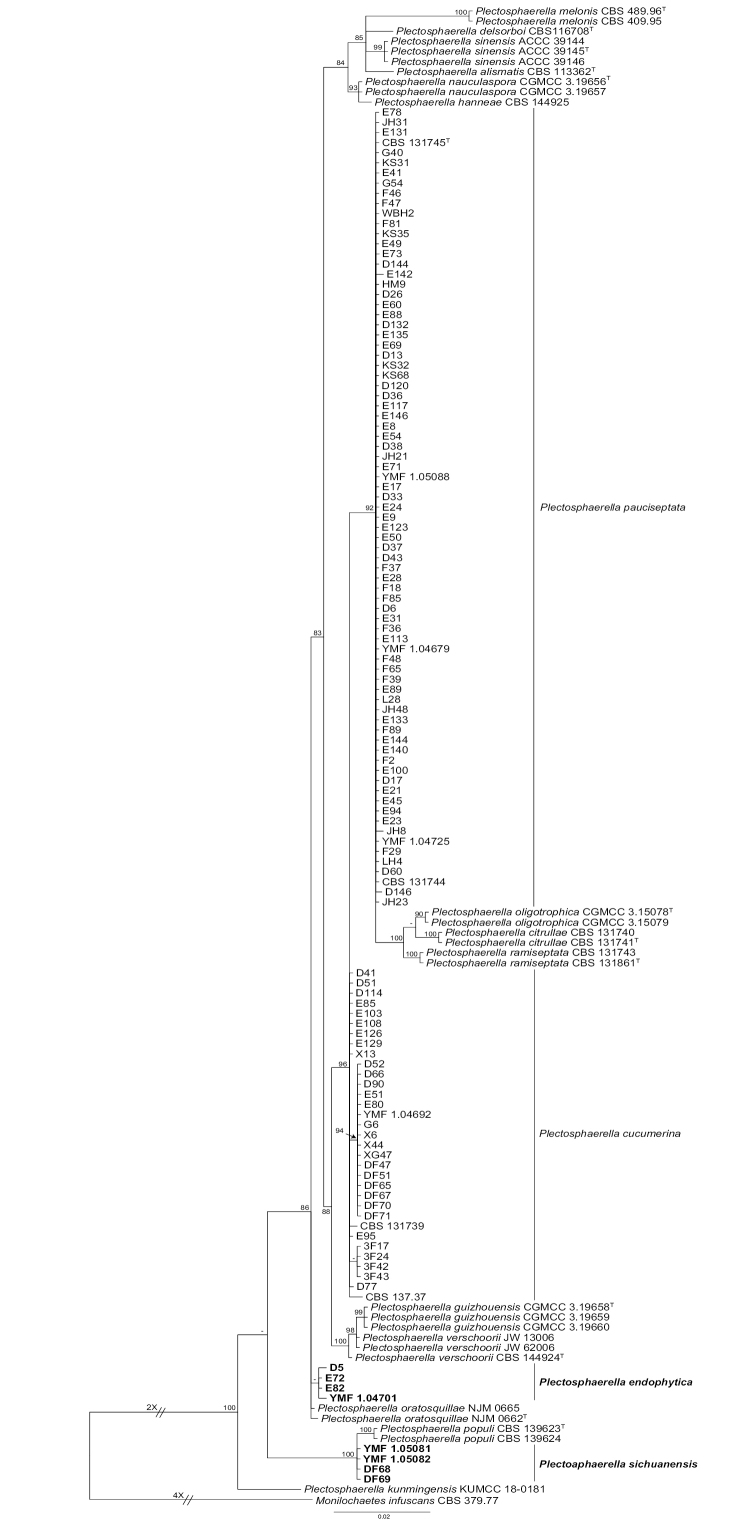
Phylogenetic tree inferred from a Bayesian analysis based on ITS sequences of 112 *Plectosphaerella* strains obtained in this study. BIPP over 80% are shown on the respective branches. The scale bar shows the expected changes per site. Two new species are given in boldface. *Monilochaetes infuscans* CBS 379.77 serves as an outgroup.

A total of 112 strains were identified as members of *Plectosphaerella* according to the BLASTn search results using the ITS sequences. At first, we carried out individual phylogenetic analyses with ITS sequences to resolve the taxonomic position of our strains using the sequences of the accepted species into *Plectosphaerella*. This tree is shown in Figure 1. The result indicated that there are 77 strains clustered together with *P. pauciseptata* A.J.L. Phillips et al. with 0.92 Bayesian posterior probability, 27 strains clustered together with *P. cucumerina* with 0.96 Bayesian posterior probability, whereas other strains formed two individual groups. Therefore, three representative strains of *P. pauciseptata*, a representative strain of *P. cucumerina*, and three representative strains of two novel taxa were chosen among the 112 strains for single-gene and combined phylogenetic analysis.

**Figure 2.**
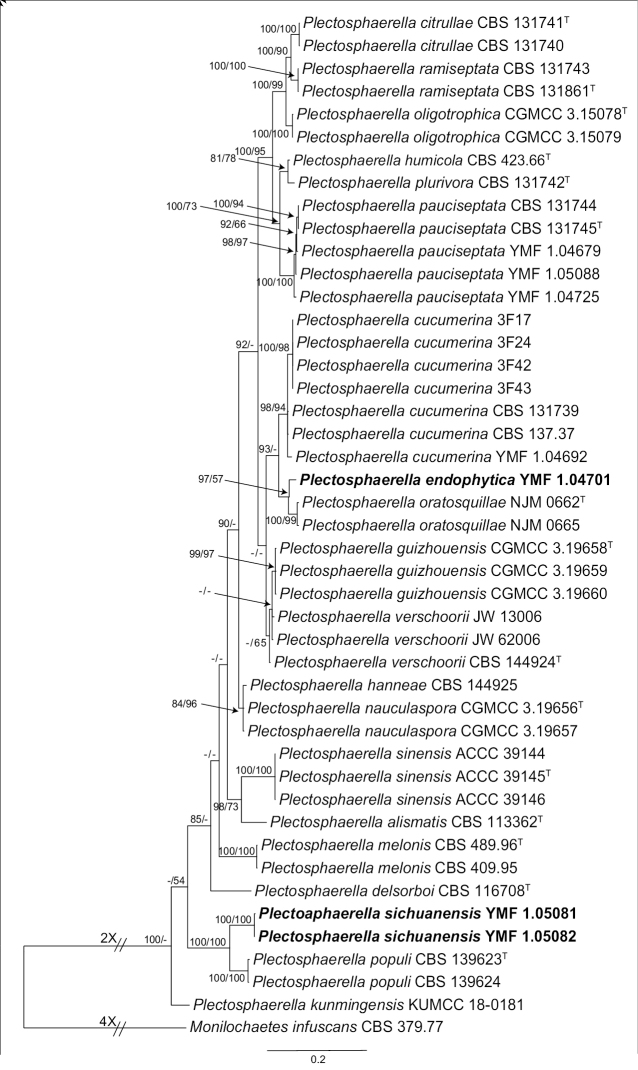
Phylogenetic tree of *Plectosphaerella* based on Bayesian analyses and Maximum Likelihood analyses of the combined sequences dataset of LSU, ITS, TEF-1α and TUB2. The numbers above branches represent BIPP (left) and MLBPs (right). BIPP over 80% and MLBPs over 50% are shown on the respective branches. The scale bar shows the expected changes per site. Two new species are given in boldface. *Monilochaetes infuscans* CBS 379.77 serves as an outgroup.

The four Bayesian trees derived from the single-gene sequence alignments (LSU, ITS, TEF-1α, TUB2) confirmed that the novel taxa were distant from other known species in *Plectosphaerella*. The Bayesian trees are available in the Suppl. material 1. The resulting combined sequence matrix included 2136 nucleotide positions (804 from LSU, 545 from ITS, 413 from TEF-1α, 374 from TUB2), with *M. infuscans* CBS 379.77 as the outgroup. The tree topology is shown in Figure 2, with the Bayesian posterior probabilities over 80% and ML bootstrap support over 50% indicated for respective clades. In this phylogenetic tree, *P. endophytica* represented by strain YMF 1.04701 was close to *P. oratosquillae* (NJM 0662 and NJM 0665) and formed a single clade with 0.97 Bayesian posterior probability and 57% ML bootstrap proportions. Similarly, *P. sichuanensis* represented by strains YMF 1.05081 and YMF 1.05082 were close to *P. populi* Ullah et al. (CBS 139623) with 1.00 Bayesian posterior probability and 100% ML bootstrap proportions. Considering distinct morphological differences, we propose to describe our isolates as two new species in *Plectosphaerella*.

### Taxonomy

#### Plectosphaerella endophytica

Taxon classificationFungiGlomerellalesPlectosphaerellaceae

Z.F. Yu & X.Q. Yang sp. nov.

86C49DCC-9026-5E0C-87F5-050F5A2F27E8

838656

Figure 3

##### Etymology.

Latin, *endophytica* meaning endophytic, growing within plant tissue.

##### Description.

Colony on CMA after 3 d, hyphae hyaline, smooth, septate, thin-walled, branched, 1.9–3.3 µm (x̄ = 2.6 μm, n = 10) wide. Conidiophores macronematous, mononematous, erect, straight or flexuous, smooth-walled, hyaline, unbranched or occasionally irregular branched, sometimes 1–2-septate. Conidiogenous cells phialides, subulate, integrated, terminal, determinate, hyaline, smooth-walled. Conidia solitary, acrogenous, broadly navicular to broadly fusiform, suboblong or ellipsoidal, 0–1-septate, usually constricted at septum, bi-guttulate, hyaline, smooth-walled, aseptate conidia abundant, 5–9.1 × 2.5–3.5 µm (x̄ = 7.8 × 3.1 µm, n = 30); septate conidia scarce, 8.8–10.1 × 3.7–4.6 µm (x̄ = 9.4 × 4.1 µm, n = 30), forming hyaline to white mucilaginous masses. Sexual morph and chlamydospores absent.

##### Culture characteristics.

Colonies on OA reaching 52 mm diameter, on PDA reaching 48 mm diameter and on CMA reaching 43 mm diameter in 14 d at 25 °C. On PDA, colonies white, dense, fluffy hyphae growth in the medium surface, outermost mycelia formed an annule, margin smooth and entire, sporulation abundant, reverse pale yellow to white.

##### Typification.

China, Yunnan Province, Kunming, The Dian Lake, 24°96'N, 102°66'E, 1886 m alt., isolated from *Hydrilla verticillata* (L.f.) Royle as an endophyte, 20 Jul. 2014, Z.F. Yu, YMF 1.04701 (Holotype), ex-type CCTCC AF 2021053.

##### Notes.

Although the phylogenetic analyses showed that our isolate *Plectosphaerella endophytica* is close to *P. oratosquillae*, the conidia of *P. oratosquillae* are aseptate, multi-guttulate (Duc et al. 2009). Furthermore, *P. endophytica* is most similar to *P. verschoorii* Hern.-Restr. & Giraldo López in the septa of conidia; both species produce 0–1-septate conidia, and septate conidia are larger than aseptate conidia (*P. verschoorii*: 1-septate conidia, 8–11.5 × 2–3 μm; aseptate conidia, 3–8.5 × 2–3 μm), but there are obvious difference in the shape of conidia, *P. endophytica* was deeply constricted at septa. Besides, the phialides of *P. verschoorii* are shorter (up to 14 μm) (Giraldo et al. 2019).

**Figure 3.**
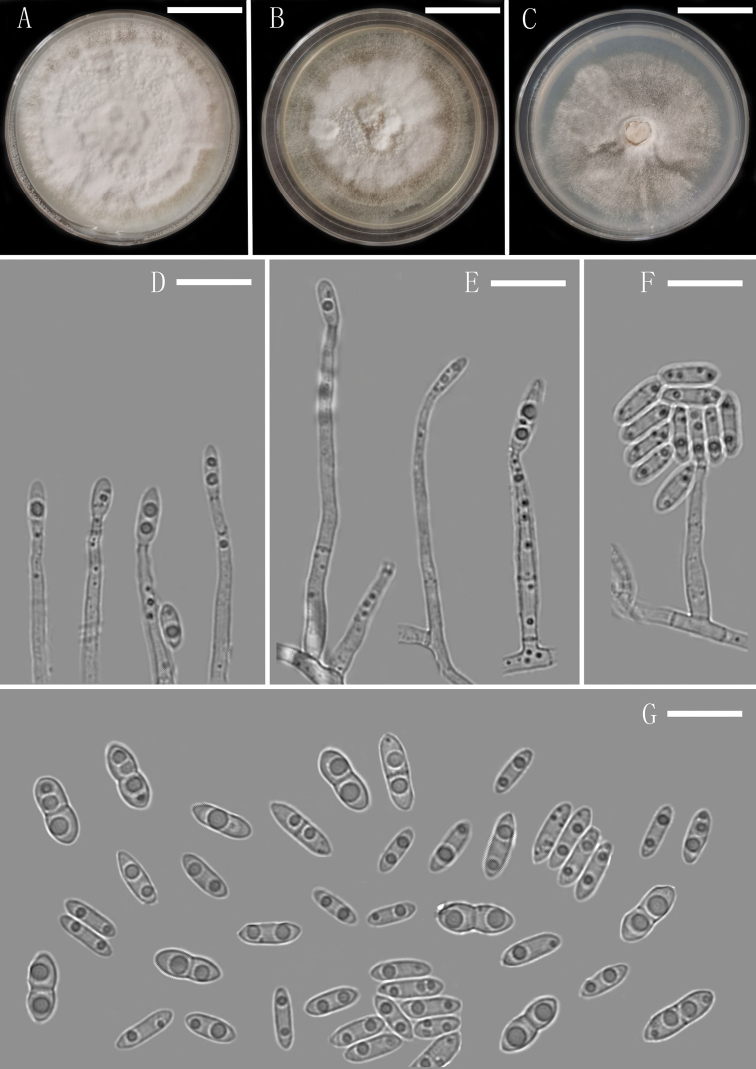
*Plectosphaerella endophytica* (YMF 1.04701, holotype) **A–C** colony on OA, PDA and CMA after 14 d **D–F** conidiophores and Phialides **G** conidia. Scale bars: 1.35 cm (**A–C**), 10 µm (**D–G**).

#### Plectosphaerella sichuanensis

Taxon classificationFungiGlomerellalesPlectosphaerellaceae

Z.F. Yu & X.Q. Yang sp. nov.

7769D6F4-C3DD-5A3F-A015-9C8B88EE9B56

838657

Figure 4

##### Etymology.

Latin, *sichuanensis*, referred to Sichuan Province, the locality where the fungus was found.

##### Description.

Colony on CMA after 3 d, vegetative hyphae hyaline, septate, commonly branched, smooth, thin-walled, mostly 2.5–3.5 μm (x̄ = 2.9 μm, n = 10) wide. Conidiophores macronematous, mononematous, erect, straight or flexuous to sinuate, hyaline, smooth, unbranched or rarely branched, aseptate. Conidiogenous cells phialides, integrated, terminal, determinate, subulate, hyaline, smooth. Conidia acrogenous, ellipsoidal, unicellular, smooth-walled, hyaline, 1–3 guttulate, 4.2–6.8 × 2.5–3.7 μm (x̄ = 5.2 × 3.3 µm, n = 30), forming hyaline to white mucilaginous masses. Sexual morph and chlamydospores absent.

##### Culture characteristics.

Colonies on OA reaching 50 mm diameter, on PDA reaching 47 mm diameter and on CMA reaching 42 mm diameter in 14 d at 25 °C. On PDA, colonies pale brown to white, flat, repressed, plicated, partly immersed, a few white aerial hyphae grew in the middle of the medium, margin regular, frontier distinct, reverse pale brown to white.

##### Typification.

China, Sichuan Province, Daofu, 30°98'N, 101°13'E, 2960 m alt., isolated from *Potamogeton pectinatus* as an endophyte, 20 Jul. 2015, Z.F. Yu, YMF 1.05081 (Holotype), ex-type CCTCC AF 2021054, another strain checked: YMF 1.05082.

##### Notes.

In the phylogenetic tree, the closest species to *Plectosphaerella sichuanensis* is *P. populi*, but *P. populi* can be distinguished from *P. sichuanensis* by its smaller aseptate conidia (Crous et al. 2015). The size and shape of conidia of *P. sichuanensis* is more similar to *P. cucumerina*, expect that *P. cucumerina* presents longer phialides (up to 69 μm) (Carlucci et al. 2012). In addition, *P. sichuanensis* resembles *P. citrullae* Carlucci et al., *P. pauciseptata* and *P. oratosquillae* in lacking septate conidia. However, it can be distinguished from *P. citrullae* by polyphialides conidiogenous cells; from *P. pauciseptata* and *P. oratosquillae* by bi-guttulate conidia (Duc et al. 2009; Carlucci et al. 2012).

**Figure 4.**
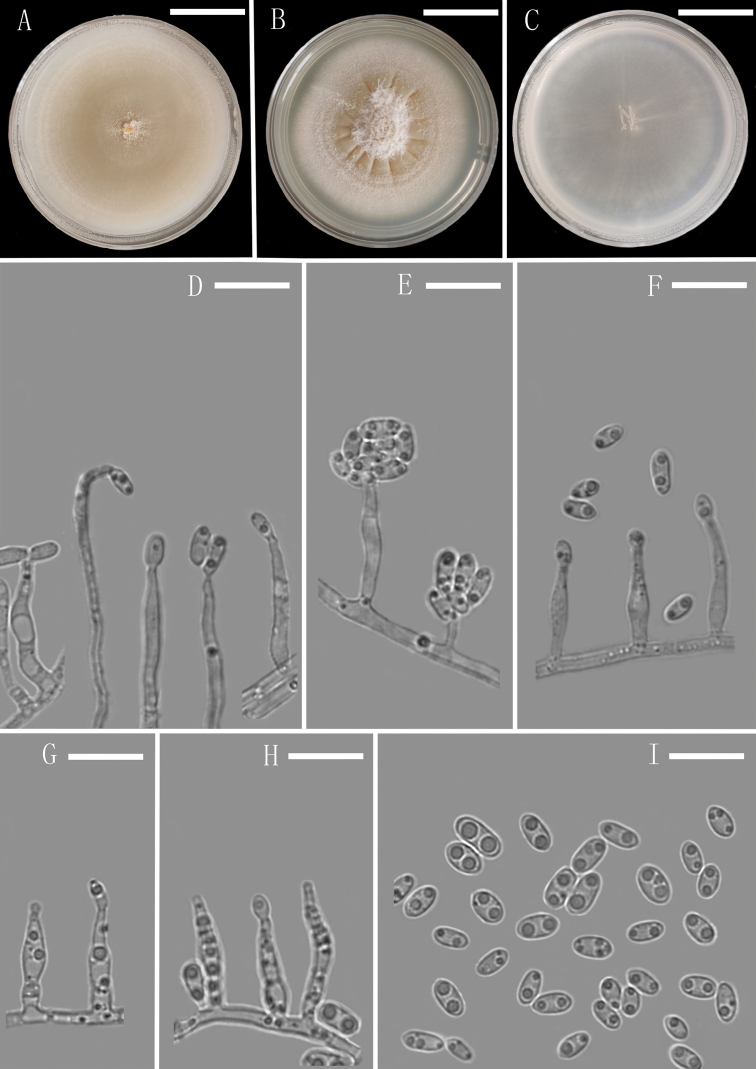
*Plectosphaerella sichuanensis* (YMF 1.05081, holotype) **A–C** colony on OA, PDA and CMA after 14 d **D–H** conidiophores and Phialides **I** conidia. Scale bars: 1.35 cm (**A–C**), 10 µm (**D–I**).

#### Plectosphaerella cucumerina

Taxon classificationFungiGlomerellalesPlectosphaerellaceae

(Lindf.) W. Gams, in Domsch & Gams

35CC9E68-95F5-593E-972A-ABCCACA61098

Figure 5

##### Description.

Colony on CMA after 3 d, hyphae hyaline, septate, smooth, thin-walled, branched, 2.5–3.5 μm (x̄ = 3.2 μm, n = 10) wide. Conidiophores macronematous, mononematous, erect or flexuous to sinuate, hyaline, smooth, branched, occasionally forming hyphal coils. Conidiogenous cells phialides, terminal, determinate, subulate. Conidia acrogenous, hyaline, unicellular, smooth-walled, oblong-ellipsoidal, 1–2 guttulate, 6.6–10.7 × 2.3–3.6 μm (x̄ = 8.7 × 3.1 µm, n = 30), forming hyaline to white mucilaginous masses. Sexual morph and chlamydospores absent.

##### Culture characteristics.

Colonies on OA reaching 55 mm diameter, on PDA reaching 48 mm diameter and on CMA reaching 44 mm diameter in 14 d at 25 °C. On PDA, colonies pale brown, repressed, flat, partly immersed, some aerial hyphae grew in the middle and margin of the medium, margin regular, reverse pale brown.

##### Strain examined.

China, Sichuan Province, Baiyu, 31°00'N, 99°41'E, 4013 m alt., isolated from *Myriophyllum spicatum* as an endophyte, 20 Aug. 2015, Z.F. Yu, YMF 1.04692.

**Figure 5.**
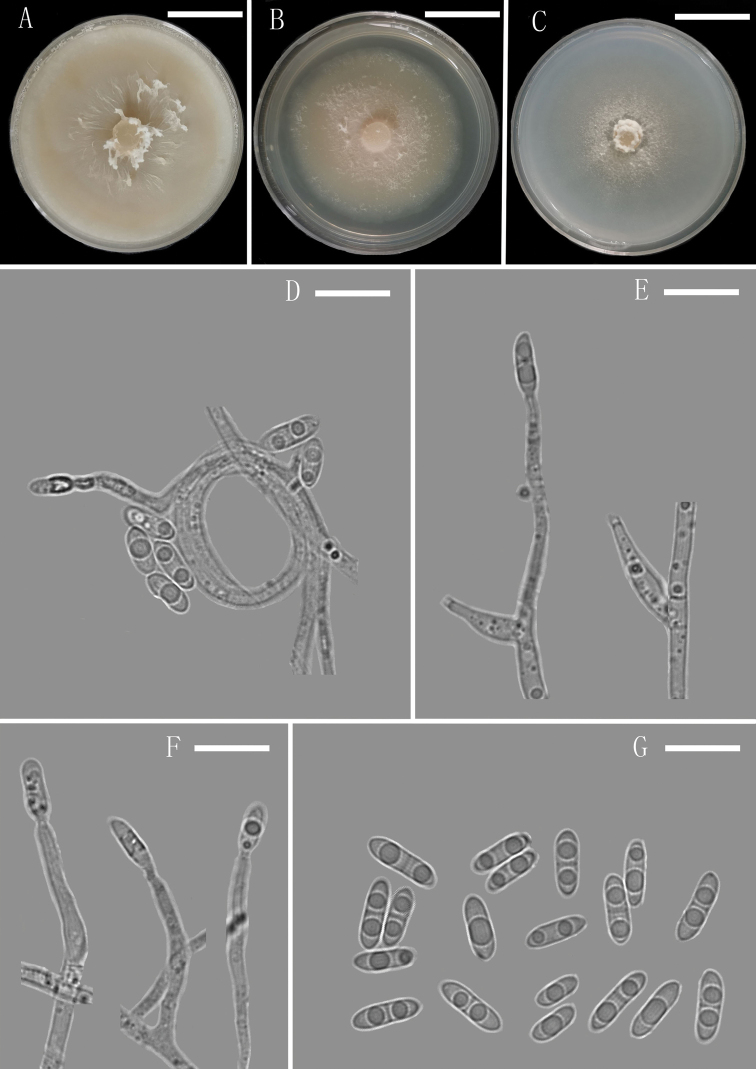
*Plectosphaerella cucumerina* (YMF 1.04692) **A–C** colony on OA, PDA and CMA after 14 d **D** hyphal coils **E, F** conidiophores and Phialides **G** conidia. Scale bars: 1.35 cm (**A–C**), 10 µm (**D–G**).

#### Plectosphaerella pauciseptata

Taxon classificationFungiGlomerellalesPlectosphaerellaceae

A.J.L. Phillips, A. Carlucci & M.L. Raimondo

DF5B5F92-F840-5A2D-B482-CE2198EBE0DA

Figure 6

##### Description.

Colony on CMA after 3 d, hyphae hyaline, septate, commonly branched, thin-walled, smooth, 2.5–3.0 μm (x̄ = 2.6 μm, n = 10) wide. Conidiophores macronematous, mononematous, erect, straight or flexuous, hyaline, smooth, aseptate, occasionally branched. Conidiogenous cells phialides, terminal, determinate, subulate, hyaline, smooth, thin-walled. Conidia acrogenous, hyaline, oblong-ellipsoidal, unicellular, smooth-walled, multi-guttulate, 5.5–12.5 × 2.5–3.5 μm (x̄ = 9.7 × 3.3 µm, n = 30), forming hyaline to white mucilaginous masses. Sexual morph and chlamydospores absent.

##### Culture characteristics.

Colonies on OA reaching 55 mm diameter, on PDA reaching 49 mm diameter and on CMA reaching 45 mm diameter in 14 d at 25 °C. On PDA, colonies white, dense, raised, aerial hyphae growth in the medium surface, margin regular, frontier distinct, reverse pale brown to white.

##### Strain examined.

China, Yunnan Province, Erhai, 25°43'N, 100°11'E, 1964 m alt., isolated from *Myriophyllum spicatum* as an endophyte, 31 Jul. 2014, Z.F. Yu, YMF 1.05088, YMF 1.04679, YMF 1.04725.

**Figure 6.**
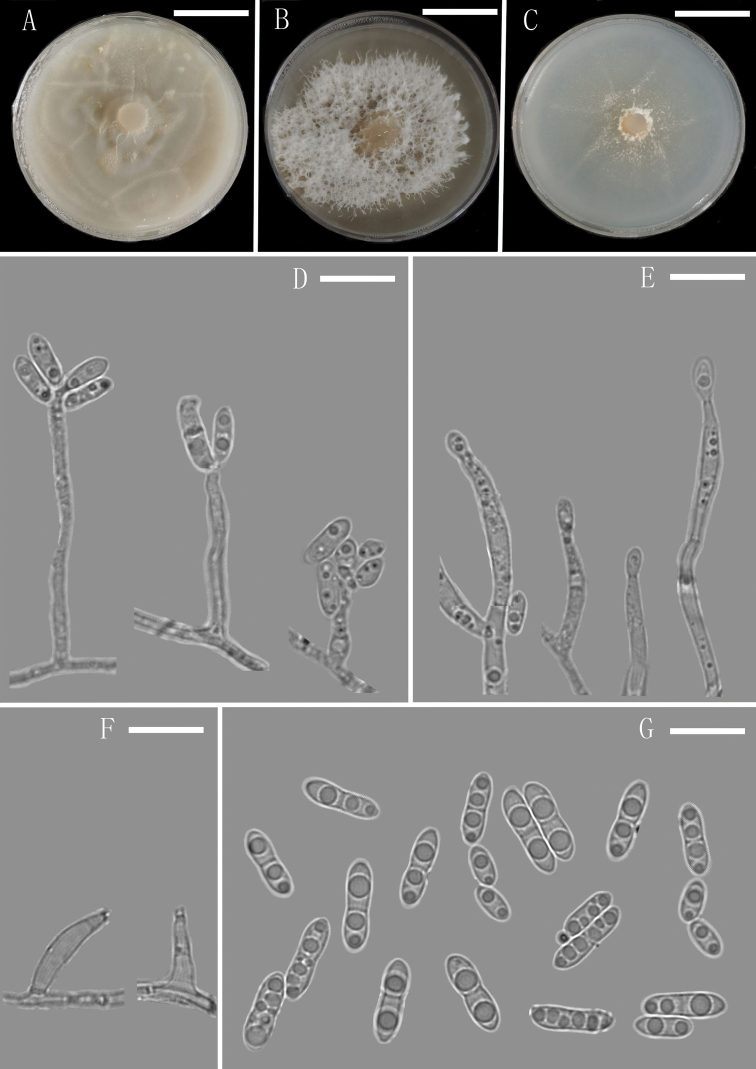
*Plectosphaerella pauciseptata* (YMF 1.05088) **A–C** colony on OA, PDA and CMA after 14 d **D–F** conidiophores and Phialides **G** conidia. Scale bars: 1.35 cm (**A–C**), 10 µm (**D–G**).

## Discussion

Sexual reproduction of *Plectosphaerella* has only been reported for three species (Giraldo and Crous 2019; Phookamsak et al. 2019). Most strains, by contrast, show asexual morphs in substrate, so the main distinguishing characteristics of this genus are based on the ratio of septate conidia, conidial dimensions and shape, absence or presence of chlamydospores (Carlucci et al. 2012; Arzanlou et al. 2013). These simple features and similar lifestyle make identification more difficult, so the classical way is no longer a valid and reliable marker (Palm et al. 1995; Zare et al. 2007; Carlucci et al. 2012). The ITS and LSU regions were considered to be a necessary condition for species identification of *Plectosphaerella* (Duc et al. 2009; Carlucci et al. 2012; Liu et al. 2013; Crous et al. 2015). However, *Plectosphaerella* species exhibit a relatively low degree of ITS or LSU molecular diversity, showing more molecular markers are needed to distinguish species (Carlucci et al. 2012). Recently, fragments of several protein-coding genes were selected to efficiently elucidate the taxonomy of this genus, including CaM, TEF-1α, TUB2 and RPB2 (Su et al. 2017; Giraldo et al. 2019; Giraldo and Crous 2019; Phookamsak et al. 2019; Zhang et al. 2019). In this study, a multi-locus analysis improved the diagnostic level of the whole genus.

In this study, among 129 sample sites in Yunnan and Sichuan province, *Plectosphaerella* species were isolated from 13 sampling sites. Regions are arranged in order of isolation frequency: Eehai (39.29%), Dian Lake (20.54%), Fuxian Lake (13.39%), Daofu (8.04%) and other places were below 5%. It seems that distribution of *Plectosphaerella* is impacted by human activities, because there are more human activities around the first three sites. In addition, *Plectosphaerella* species only occur in six aquatic plants including *Batrachium*, *Halerpestes*, *Hippuris*, *Hydrilla*, *Myriophyllum*, and *Potamogeton*, although we investigated 30 aquatic plants. So *Plectosphaerella* species exhibit medium host diversity and relatively narrow geographic distribution when aquatic plants serve as hosts.

Secondly, the separation frequency of *Plectosphaerella pauciseptata* was the highest at 68.78%, followed by *P. cucumerina* at 24.11%, and two new species were the lowest at 3.57%. According to this data and previous studies, *P. cucumerina* and *P. pauciseptata* are still the most widely distributed species either on land or within aquatic plants, although *P. pauciseptata* is more common in aquatic plants. On the other hand, only four species were identified among 112 strains. One reason may be that species diversity of *Plectosphaerella* is low within aquatic plants. Other reasons may be *Plectosphaerella* species exhibit a relatively low degree of ITS molecular diversity. Based on this, perhaps we treat some different species as two known species, which reduce the diversity of *Plectosphaerella*. Regrettably, we did not preserve all strains except for representative strains. Or else, we should sequence other loci of all strains, especially for two species, *P. pauciseptata*, *P. cucumerina*, more loci are needed to distinguish them.

This study revealed *Plectosphaerella* species within aquatic plants from the southwest of China, although only 112 strains and four species were reported, which also enrich the ecology and diversity of the genus. However, our survey was limited, hence it is necessary to know if our result is concordant with those of other regions. So in-depth work is required to obtain a more integrated knowledge of their biodiversity and distribution in water bodies.

## Supplementary Material

XML Treatment for Plectosphaerella endophytica

XML Treatment for Plectosphaerella sichuanensis

XML Treatment for Plectosphaerella cucumerina

XML Treatment for Plectosphaerella pauciseptata

## Supplementary materials

Supplementary material 1 Figures S1–S4 Data type phylogenetic tree Explanation note **Figure S1.** Phylogenetic tree generated by Bayesian inference based on sequences of the ITS. Monilochaetes infuscans CBS 379.77 serves as an outgroup. Bayesian posterior probability over 80% are shown at the nodes. Two new species are given in boldface. **Figure S2.** Phylogenetic tree generated by Bayesian inference based on sequences of the LSU. Monilochaetes infuscans CBS 379.77 serves as an outgroup. Bayesian posterior probability over 80% are shown at the nodes. Two new species are given in boldface. **Figure S3.** Phylogenetic tree generated by Bayesian inference based on sequences of the TEF-1α. Bayesian posterior probability over 80% are shown at the nodes. Two new species are given in boldface. **Figure S4.** Phylogenetic tree generated by Bayesian inference based on sequences of the TUB2. Bayesian posterior probability over 80% are shown at the nodes. Two new species are given in boldface. https://binary.pensoft.net/file/541316This dataset is made available under the Open Database License (http://opendatacommons.org/licenses/odbl/1.0/). The Open Database License (ODbL) is a license agreement intended to allow users to freely share, modify, and use this Dataset while maintaining this same freedom for others, provided that the original source and author(s) are credited.Xiao Qian Yang, Shi Yun Ma, Ze Xiang Peng, Zhong Qiao Wang, Min Qiao, Zefen Yu
