# Supplementary material for: Diversity of Plectosphaerella within aquatic plants from southwest China, with P. endophytica and P. sichuanensis spp. nov
Source: MycoKeys. 2021 May 11;80:57–75. doi: 10.3897/mycokeys.80.64624 (PMC8131346; doi:10.3897/mycokeys.80.64624)
Supplement: Supplementary material 1 — Figures S1–S4 [file mycokeys-80-057-s001.zip › Supplementary Material/Legends for supplementary figures.docx]

Figure 1. Phylogenetic tree generated by Bayesian inference based on sequences of the ITS. *Monilochaetes infuscans* CBS 379.77 serves as an outgroup. Bayesian posterior probability over 80% are shown at the nodes. Two new species are given in boldface.

Figure 2. Phylogenetic tree generated by Bayesian inference based on sequences of the LSU. *Monilochaetes infuscans* CBS 379.77 serves as an outgroup. Bayesian posterior probability over 80% are shown at the nodes. Two new species are given in boldface.

Figure 3. Phylogenetic tree generated by Bayesian inference based on sequences of the TEF-1α. Bayesian posterior probability over 80% are shown at the nodes. Two new species are given in boldface.

Figure 4. Phylogenetic tree generated by Bayesian inference based on sequences of the TUB2. Bayesian posterior probability over 80% are shown at the nodes. Two new species are given in boldface.
