# Supplementary figures and images for: Diversity of Plectosphaerella within aquatic plants from southwest China, with P. endophytica and P. sichuanensis spp. nov
Source: MycoKeys. 2021 May 11;80:57–75. doi: 10.3897/mycokeys.80.64624 (PMC8131346; doi:10.3897/mycokeys.80.64624)

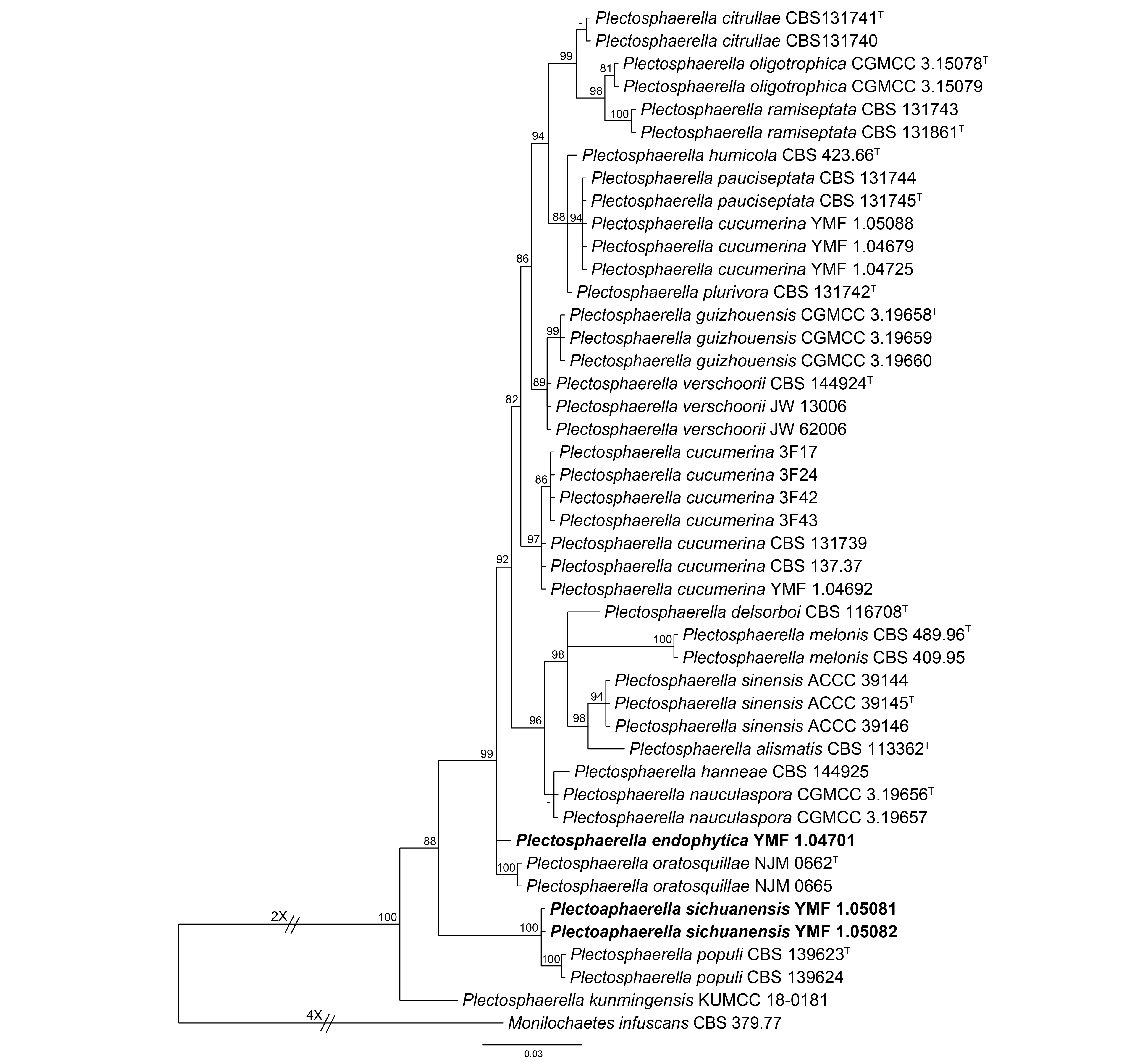

Supplement: Supplementary material 1 — Figures S1–S4 [file mycokeys-80-057-s001.zip › Supplementary Material/Supplementary Figure 1.jpg]

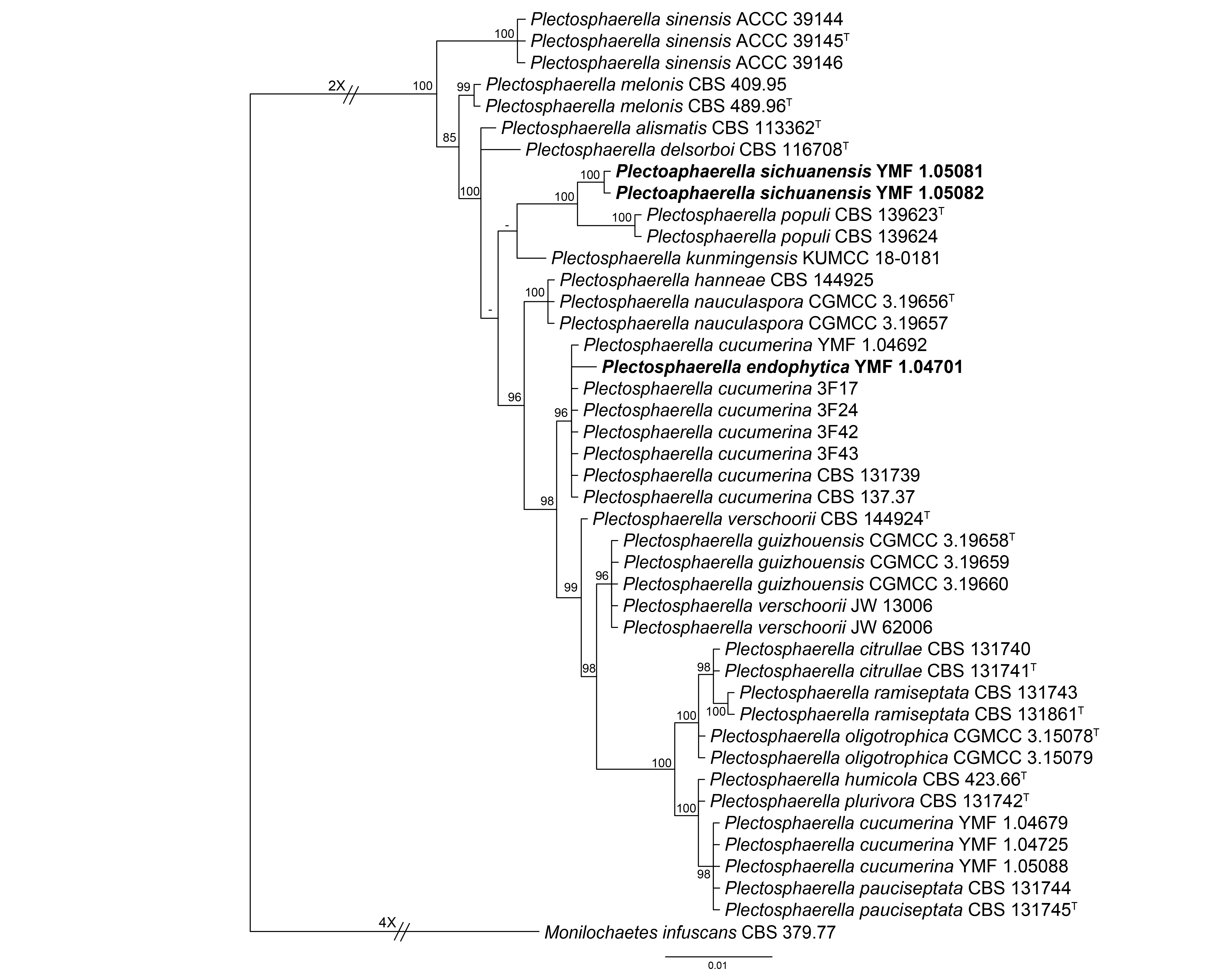

Supplement: Supplementary material 1 — Figures S1–S4 [file mycokeys-80-057-s001.zip › Supplementary Material/Supplementary Figure 2.jpg]

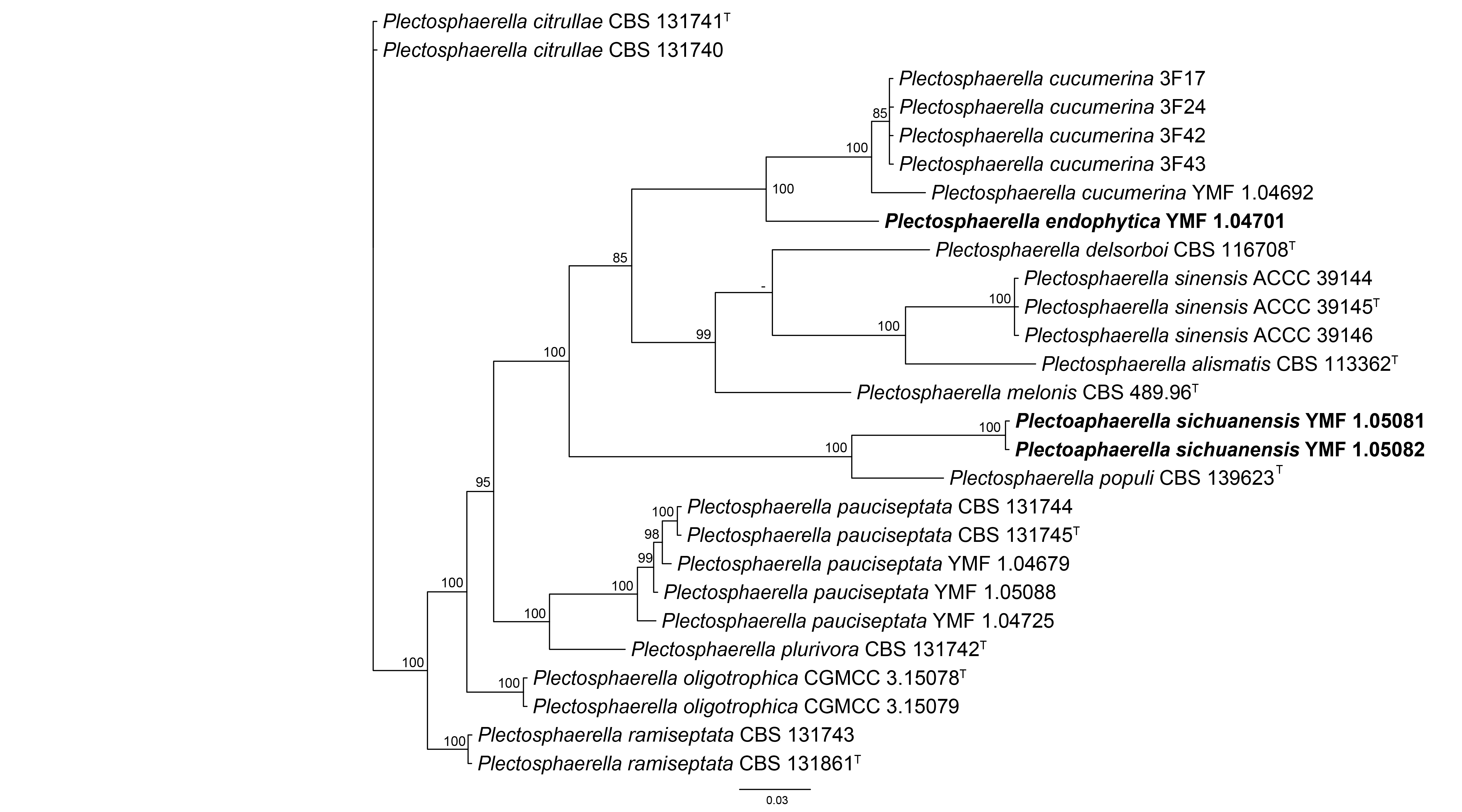

Supplement: Supplementary material 1 — Figures S1–S4 [file mycokeys-80-057-s001.zip › Supplementary Material/Supplementary Figure 3.jpg]

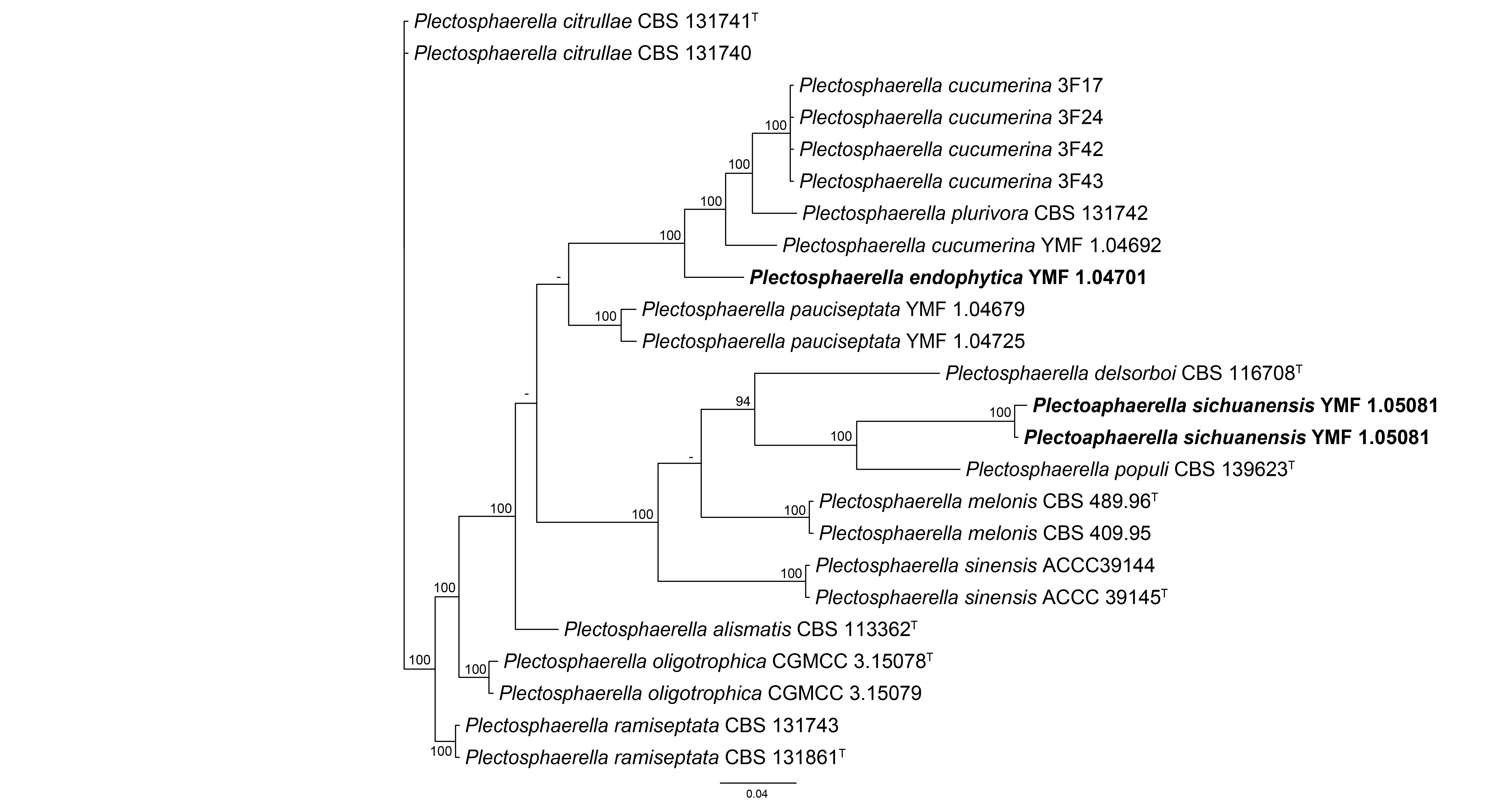

Supplement: Supplementary material 1 — Figures S1–S4 [file mycokeys-80-057-s001.zip › Supplementary Material/Supplementary Figure 4.jpg]
